# Factors associated with completeness in documentation of diagnostic work-up and treatment in patients with breast cancer in Sudan

**DOI:** 10.3332/ecancer.2023.1632

**Published:** 2023-11-17

**Authors:** Noon I Eltoum, Nicole E Caston, Lily Gutnik, Mahmoud A Alfardous Alazm, Feras O Mohamed, Lama M Abdalkarem, Saad A S Ali, Abrar Z Badawi, Nicole L Henderson, Andres Azuero, Gabrielle Rocque

**Affiliations:** 1 Faculty of Medicine, University of Medical Sciences and Technology, PO Box 12810, Khartoum, Sudan; 2 Department of Hematology and Oncology, University of Alabama at Birmingham, 1808 7th Avenue South, Birmingham, AL 35233, USA; a https://orcid.org/0000-0003-4210-8012

**Keywords:** breast cancer, sub-Saharan Africa, receptor status, incomplete documentation

## Abstract

**Purpose:**

This study evaluates the relationship between geography and ethnicity on the completeness of documentation of diagnostic work-up and treatment modalities in Sudan for patients with breast cancer.

**Methods:**

This retrospective study used data abstracted from patients with breast cancer receiving cancer care at Sudan’s largest cancer centre (Radiation and Isotopes Center Khartoum) in 2017. Patient demographic and clinical characteristics were abstracted from paper medical records. Odds ratios and 95% confidence intervals were estimated to evaluate complete diagnostic work-up on ethnic group, origin and residence using binomial logistic regression models.

**Results:**

Of 237 patients, the median age was 52 (interquartile range 43–61). Most often patients identified as Arab (68%), originated from Central, Northeastern and Khartoum regions (all 28%) and lived in the Khartoum region (52%). Overall, 49% had incomplete diagnostic work-up, with modest differences by ethnicity and geography. In adjusted analyses, non-statistical differences were found between the ethnic group, geographic origin and residence and having complete diagnostic work-up. For treatment modality, significant differences were observed between receptor status and receiving hormone therapy (*p* = 0.004). Only 28% of patients with HR+ breast cancer received hormonal therapy. For those with HR− or undocumented breast cancer subtype, 36% and 17% received hormone therapy, respectively.

**Conclusion:**

Approximately half of Sudanese patients with breast cancer had incomplete diagnostic work-up, irrespective of ethnicity and geography. Moreover, a high proportion of patients received inappropriate treatment. This underlines a considerable systems-based quality gap in care delivery, demanding efforts to improve diagnostic work-up for all patients with breast cancer in Sudan.

## Introduction

In many low-resource countries, breast cancer staging and testing for receptors status is inconsistent, resulting in treatment below the standard of care [1–4]. Most radiologic equipment that is needed for staging cancers is only available in major institutions across sub-Saharan Africa (SSA) and others, like bone scintigraphy, are fairly uncommon across the board [4]. A pilot survey conducted in SSA of cancer facilities that were members of the African Organisation for Research and Training in Cancer (AORTIC) found that computed tomography scans and magnetic resonance imaging were only available in 79% and 58%, respectively [4]. Furthermore, previous work in SSA documented that testing for the key biomarkers needed for selecting treatment, oestrogen receptor (ER), progesterone receptor (PR) and human epidermal growth factor-2 (HER2) receptor status is expensive and often inaccessible for many patients and providers [2, 3, 5, 6]. A study conducted in Sudan in 2010, reported that ER and PR status results were only available in 4% of patients [3]. These issues can be compounded by a lack of high-quality medical documentation in many hospitals due to the absence of electronic documentation systems, unreliable handwritten information and poor storage of files. This lack of access to and documentation of appropriate diagnostic tests may lead to suboptimal staging and overall management of breast cancer in under-resourced countries [4].

Access issues are often aggravated by other country-specific challenges in under-resourced countries, such as Sudan. Since its independence in 1956, Sudan has faced decades of harsh climates, famine, war and political and economic instability [7]. These unstable conditions have had the most impact on healthcare quality and access, ultimately resulting in limited resources, inadequate workforce, unreliable health record system and poor implementation of health policies and programs [7]. Lack of access to health services can also be attributed to the centralisation of healthcare in the capital of Khartoum and the inequitable distribution of health facilities and health workforce between other states. Furthermore, the history of conflict between regions and ethnic subgroups of Sudan has manifested into the systemic marginalisation of certain groups of people. Ethnicity in Sudan is complex and incorporates overlapping factors related to place of origin, language, tribe, genealogy and shared history [8]. There are approximately 600 tribes that speak over 115 languages; however, nearly 70% of Sudanese people identify as being ethnically Arab, with the other 30% distributed between the Beja, the Nubian, the Fur, the Zaghawa and the Nuba [8–11]. Little is known about how language barriers and cultural differences impact receipt of breast cancer diagnostic testing and treatment. We hypothesise that differences in diagnostic work-up and appropriate treatment would be observed for different geographical and ethnic groups. Thus, this study aimed to understand the relationship between geography and ethnic group and the documentation of breast cancer receptor testing and staging in patients in Sudan and the appropriateness of treatment based on receptor status and staging.

## Methods

### Study design

This retrospective study included patients with breast cancer receiving services at Radiation and Isotopes Center Khartoum (RICK) in 2017. RICK is one of two cancer centres in Sudan, where 80% of patients with cancer receive their medical care [12]. The study population included all patients with breast cancer admitted to RICK in 2017. Data from patient records were abstracted from handwritten patient medical records. For the analysis, ethnic groups (Arab, Hausa/Fulani, Beja, Nubian, Nuba, Darfurians and other) were categorised as Arab and non-Arab, while non-Sudanese patients, male patients and those with missing demographics were excluded. Institutional Review Board approval was obtained from the management of RICK. Ethical considerations were sought from the research technical and ethical committee of the Faculty of Medicine, University of Medical Sciences and Technology (UMST) in Khartoum, Sudan.

### Patient demographics and clinical characteristics

Patients’ age at diagnosis and breast cancer stage were obtained from the patient’s medical record (paper-based records).

### Outcomes

#### Availability of breast cancer receptor status and staging

Pathology reports and patient medical records were used to determine documentation of staging and immunohistochemical tests for ER, PR and HER2 receptor status. If available, tumour-node-metastasis classification of breast cancer was obtained from the patient’s file and then grouped into the overall anatomic stage using the American Joint Committee on Cancer (AJCC) staging reference [13]. For breast cancer subtypes, immunohistochemical tests were used to group patients into having HR+/HER2−, HR−/HER2−, HR+/HER2+ or triple-negative breast cancer. Patients were grouped into two groups: having complete diagnostic work-up (i.e., having available receptor testing and staging in their medical record) or having incomplete diagnostic work-up (i.e., missing receptor testing, staging or both).

#### Breast cancer treatment

Treatment was extracted from patient records and classified as surgery (i.e., mastectomy or lumpectomy with or without axillary lymph node dissection), chemotherapy, hormone therapy, radiation therapy and treatment not available (i.e., not administered or not documented).

### Exposures

#### Regional and ethnic distribution

**Figure 1.**
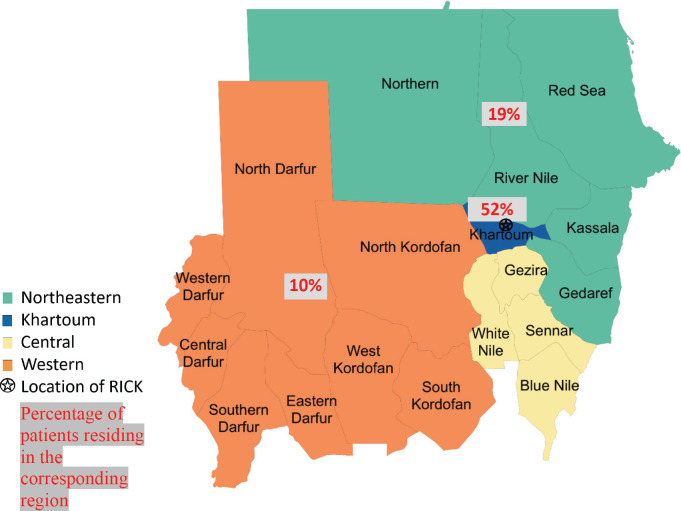
Map of Sudan showing the 18 states and the different geographic regions with the distribution of patient residence.

State of residence, state of origin, and tribe were abstracted from medical record data. States were clustered into groups based on their geographical region in Sudan (Northeastern, Central, Western and Khartoum regions) (Figure 1). Khartoum, the capital of Sudan which lies in the heart of the country, was not included in the Central region and was grouped as an independent region due to its large population and major role in the health system in Sudan; it is also where RICK is located. Patient’s tribe was self-declared, and for descriptive purposes, these tribes were classified into ethnic groups. Due to the complexity of ethnicity in Sudan and a lack of clear ethnic categories, we used ethnic categories used in previous studies that studied the genetics of Sudanese ethnic groups [10, 11]. For our study, we adopted the ethnic classification used by Dobon *et al* [10] and Babiker *et al* [11], researchers who focused on the genetic variation and population structure of Sudanese people. Patients were grouped into broader ethnic categories, including Arab, Beja, Hausa/Fulani, Nubian, Nuba, Darfurians, and other. Hausa and Fulani groups were grouped together due to small sample sizes and ethnic similarity. Additionally, although we recognise ‘Darfurian’ is not an ethnic group, Dobon *et al* [10] categorised non-Arab tribes of West Sudan into one ethnic category called ‘Darfurian’ due to their shared socio-economic activity, geographic region, and linguistic family [10].

### Statistical analysis

Descriptive statistics were calculated using frequencies and percentages for categorical variables and median and interquartile range (IQR) for continuous variables. Percentages of odds ratios (OR) and 95% confidence intervals (CI) were estimated to evaluate complete diagnostic work-up on ethnic group, origin and residence using binomial logistic regression models. Models were adjusted for age. The alpha level was set at 0.05 and differences of at least 0.1 in proportions were considered clinically important. Analyses were performed using SAS© software, version 9.4 (SAS Institute, Cary, NC, USA).

## Results

### Patient characteristics

Of 237 female patients with breast cancer included, the median age was 52 years (IQR 42–61). Patients were most often Arab (68%); 17% of patients were Nubian, 8% were Darfurian, 3% were Beja, 3% were Hausa/Fulani and 2% were Nuba. The majority of patients originated from the Central and Northeastern regions (28%), followed by Khartoum (27%) and 16% of patients originated from the Western region. Most patients lived in the Khartoum region (52%); 19% of patients lived in the Central and Northeastern regions; and 10% of patients lived in the Western region (Table 1).

### Diagnostic work-up

Approximately half of patients had complete diagnostic work-up (51%); 20% of patients were missing receptor testing and 21% were missing staging; 8% of patients were missing both receptor testing and staging. The mean age of patients with complete diagnostic work-up (52) was similar to the mean age of patients with incomplete diagnostic work-up (52) (*t* = −0.05, *p* = 0.958).

After adjusting for age, non-Arab patients had 28% higher odds of having a complete diagnostic work-up when compared to Arab patients, however, this was not statistically significant (OR 1.28; 95% CI 0.74–2.22; Table 2). In adjusted analyses, when compared to patients originating from Khartoum, patients originating from the Central region had 37% lower odds of having a complete diagnostic work-up (OR 0.63; 95% CI 0.47–1.86). Also when compared to patients originating from Khartoum, patients originating from the Western region and the Northeastern region had 15% and 6% lower odds of having complete diagnostic work-up, respectively (OR 0.85; 95% CI 0.38–1.88) (OR 0.94; 95% CI 0.47–1.86). However, these observed differences are not statistically significant. When compared to patients residing in Khartoum, patients residing in the Central and Western regions had 38% lower odds of having complete diagnostic work-up which was not statistically significant (OR 0.62; 95% CI 0.31–1.23) (OR 0. 62; 95% CI 0.25–1.53). Patients living in the Northeastern region had 15% lower odds of having complete diagnostic work-up when compared to patients living in Khartoum (OR 0.85; 95% CI 0.43–1.68). These observed differences are not statistically significant.

### Staging

Of 237 patients, 29% had missing documentation of staging. Among all patients, stage III breast cancer was the most common (37%), followed by stage II and stage IV (14% each). Stage I was the least common (6%).

### Receptor status

Overall, 29% of patients did not have documentation of receptor status. Amongst those with receptor status available, HR+/HER2− (30%) breast cancer was the most common subtype. Triple-negative breast cancer is the second most frequent subtype at 19%; 12% of patients had HR+/HER2+ and 11% had HR−/HER2+ breast cancer (Table 3).

### Treatment modality

Majority of patients (90%) had documentation of treatments prescribed or received. The most common treatment modality was chemotherapy alone (41%), followed by the combination of surgery and chemotherapy (11%), the combination of chemotherapy and hormone therapy (9%), hormone therapy alone (7%), the combination of chemotherapy and radiation therapy (5%), surgery alone (4%) and the combination of surgery, chemotherapy and hormone therapy (4%).

Significant differences were observed between receptor status and receiving surgery (*p* = 0.013), but not between stage and receiving surgery (*p* = 0.166) (Table 4). When compared to patients who didn’t receive surgery, 40% of patients with TNBC, 30% of patients with HR+/HER2− disease, 29% of patients with HR+/HER2+ disease, 16% of patients with HR−/HER2+ disease and 13% of patients without receptor status documentation received surgery.

Additionally, significant differences were observed between receptor status and receiving hormone therapy (*p* = 0.004). Among patients with HR+ breast cancer, only 28% received hormonal therapy. For those with HR− or undocumented breast cancer breast cancer subtype, 36% and 17% received hormone therapy, respectively. Differences observed between other chemotherapy and radiotherapy and receptor status and staging were also not significant.

## Discussion

Our study suggests that there are widespread gaps in the completion of diagnostic work-up (i.e., both receptor testing and staging) among all patients with breast cancer in Sudan, irrespective of geography and ethnicity. Overall, 49% of patients had incomplete breast cancer diagnostic work-up; 20% of patients were missing receptor status; 21% of patients were missing staging, and 8% were missing both. While considerably lower than the standard of care, these findings closely align with research on other cancers from the African region. It is important to highlight that there is a limited body of research addressing unstaged breast cancer in SSA. Nevertheless, a population-based study by Seraphin *et al* [14] within the same geographical context revealed that 47% of patients with prostate cancer were unstaged, and most patients with incomplete documentation did not receive diagnostic workup or treatment. In contrast, a study that assessed the New Zealand Cancer registry, found that 12.3% of patients with breast cancer in New Zealand are unstaged [15]. In the US, there has been a sharp decline in unstaged cancers, however, several studies have suggested that increased levels of unstaged breast cancer are seen with increasing age, patients of racial/ethnic minorities [16, 17], and patients from rural areas [18]. In contrast to our hypothesis that ethnicity and geographic residence and origin would impact diagnostic work-up among Sudanese patients, non-statistical differences were observed, suggesting the role these factors play on the availability of receptor testing and staging are minor compared to overall quality gaps within the populations.

A possible significant contributing factor to the gap in recommended diagnostic work-up and lack of access is poverty secondary to the political and economic instability that has plagued Sudan since its independence. Approximately half of the Sudanese population lives below the national poverty line [19]. High out-of-pocket expenses for health services have impacted their access and utilisation. Additionally, a limited number of healthcare workers, poor communication between clinicians and labs impact cancer care in the region [20]. The absence of a well-established electronic health system and inadequate health facilities also further complicate quality healthcare delivery [20].

Our study found that hormone receptor status did not fully guide the delivery of hormone therapy. In fact, only 28% of patients who had HR+ disease received hormone therapy. On the other hand, 36% of patients who had HR disease received hormone therapy, which has been proven to be ineffective since HR− breast cancer does not respond to hormone therapy [21, 22]. One possible explanation for the considerable number of patients with HR – disease who received hormone therapy could be a delay in diagnosis. It is conceivable that these patients may have been treated blindly initially due to barriers to pathological receptor testing and alternative affordable therapies, and then subsequently found to have hormone negative disease. However, due to the nature of the incomplete handwritten medical records this information along with any treatment changes may have not been documented. Follow-up and regular monitoring of treatment effectiveness is an additional challenge, especially for patients with low income and those traveling from rural parts of Sudan. This may lead to the continuation of hormone therapy despite it being ineffective. Additionally, 17% of patients who did not have documentation of receptor testing received hormone therapy, suggesting either a lack of documentation of pathology results or inappropriate treatment administration. These findings were consistent with prior studies showing that deficiencies in diagnostic work-up have impelled clinicians in Sudan to blindly administer therapy irrespective of a patient’s receptor status and staging, leading to a decrease in the quality of cancer care [1, 3–5, 23]. Studies in other SSA countries have also demonstrated similar findings. In a hospital-based study conducted in Zimbabwe, 67% of patients were treated without knowledge of their hormone receptor status [5]. Ultimately, adequate receptor testing is needed to maximise both patients receiving appropriate therapy and avoidance of ineffective therapy for those unlikely to benefit.

Furthermore, our study found low rates of radiotherapy (11%), which is consistent with a different study that found only ten radiation therapy machines across the four comprehensive cancer centres in Sudan [24]. Breast cancer is often treated with more than one treatment modality, however, in our study, the main treatment modality physicians resorted to was chemotherapy alone (41%), followed by the combination of surgery and chemotherapy (11%) and the combination of chemotherapy and hormone therapy (9%) (Table 3). Although chemotherapy is the most prescribed treatment for Sudanese patients with breast cancer, our study found a lack of statistical differences across stages, suggesting a lack of tailoring to the risk of disease recurrence (Tables 4 and 5). It is imperative to highlight the importance of tailored, targeted therapy in enhancing overall outcomes and survival rates of patients with breast cancer, especially in those with HR + disease. Furthermore, it is crucial to recognise the potential benefits of radiotherapy in local control, decreasing recurrence and improving survival.

To address these issues, we suggest interventions that aim to establish sufficient cancer centres with appropriate and high-quality services in Sudan and SSA. However, sustaining expensive radiologic equipment in complex environments with inadequate electricity supply and poor maintenance is a huge challenge [25]. Thus, global support and governmental funding allocations must help with running costs and long-term maintenance of radiologic equipment to allow patients with breast cancer to have access to affordable radiologic imaging. This could expand diagnostic capabilities and therefore, improve cancer outcomes among patients in SSA. Additionally, since pathology testing in Sudan is inaccessible and often suboptimal, funding allocations for instituting reliable labs for pathologic testing is essential. There is also a need for educating and training physicians and healthcare workers on appropriate medical documentation. Finally, transitioning from paper-based medical records to an electronic health system will also bridge gaps in medical documentation and interdisciplinary communication.

This study has limitations. One limitation of this study is the use of data from 2017. Nonetheless, due to factors like the 2019 Revolution and the COVID-19 pandemic which have both adversely affected healthcare in Sudan, there is not anticipated to be substantial improvement in medical documentation or cancer care delivery. Additionally, many patients could have never received a diagnosis of cancer and died without seeking medical care. Another limitation is that ethnicity in Sudan is not well studied, and the usage of broader groups to categorise patients may have blunted the results of the study. Our study is also subject to information bias since data was abstracted from handwritten paper-based medical records. Due to insufficiency of medical documentation, there was no way to confirm if more pathologic testing and radiologic imaging were completed. Also, reasons for not getting diagnostic work-up were not documented in patient files and were therefore not evident. Finally, the reasons that physicians selected treatments, and the available resources for individual patients were not available for this analysis. Further work is needed to understand if practice patterns contradictory to guidelines (e.g., hormone therapy for HR breast cancer) are due to a lack of available systemic therapies or to other potential intervening factors.

## Conclusion

A high proportion of Sudanese patients with breast cancer have deficient diagnostic work-up including missing receptor testing and staging, which limits cancer care delivery, and ultimately contributes to inadequate or inappropriate treatment, which may result in poor survival. Gaps are country-wide and not isolated to specific geographical or ethnic groups. Further work is needed to understand the reasons for these deficiencies and to develop interventions to improve diagnostic work-up and treatment in low-income countries such as Sudan.

## Conflicts of interest

Dr Rocque received research funding from Genentech, Pfizer and Carevive and consulting fees for Genentech and Pfizer. All other authors declare they have no conflicts of interest.

## Funding

All other authors declare that no funds, grants or other support was received during the preparation of this manuscript.

## Author contributions

All authors contributed to the study conception and design. Material preparation and data collection were performed by MA, LA, SA, AB and NE. Analysis was performed by NC and NE and revised by AA. The first draft of the manuscript was written by NE. All authors commented on previous versions of the manuscript. All authors read and approved the final manuscript.

## Data availability

The data generated during and analysed during the study are not publicly available due to possible breach in patient confidentiality but are available from the corresponding author on reasonable request.

## Figures and Tables

**Table 1.** Demographic and clinical characteristics of sample population (*N* = 237).

		Receptor testing and staging available, *n* = 121	Missing receptor testing, *n* = 48	Missing staging, *n* = 50	Both missing, *n* = 18	Total, *N* = 237
**Ethnic group**		***n* (%)**	***n* (%)**	***n* (%)**	***n* (%)**	***n* (%)**
Arab	Arab	79 (65)	32 (67)	36 (22)	14 (78)	161 (68)
Non-Arab	Beja	4 (3)	3 (6)	0	0	7 (3)
	Darfurian	10 (8)	2 (4)	4 (8)	2 (11)	18 (8)
	Hausa/Fulani	3 (2)	4 (8)	0	0	7 (3)
	Nuba	2 (2)	1 (2)	1 (2)	0	4 (2)
	Nubian	23 (19)	6 (13)	9 (18)	2 (11)	40 (17)
Geographic origin						
	Central	29 (24)	15 (31)	17 (34)	5 (28)	66 (28)
	Khartoum	36 (30)	14 (29)	13 (26)	2 (11)	68 (28)
	Northeastern	36 (30)	12 (25)	14 (28)	5 (28)	67 (28)
	Western	20 (17)	7 (15)	6 (12)	6 (33)	39 (16)
Geographic residence						
	Central	20 (17)	10 (21)	12 (24)	4 (22)	46 (19)
	Khartoum	68 (56)	25 (52)	25 (50)	5 (28)	126 (52)
	Northeastern	23 (19)	9 (19)	9 (18)	4 (22)	45 (19)
	Western	10 (8)	4 (8)	4 (8)	5 (28)	23 (10)

^a^Non -Sudanese patients and patients with missing data for demographics were excluded

**Table 2.** Model-estimated odds ratios, predicted probabilities, and 95% CIs evaluating complete diagnostic workup (*N* = 237).

	OR (95% CI)	PP (95 CI %)
Ethnic group		
Non-Arab	1.28 (0.74, 2.22)	0.55(0.44, 0.66)
Arab	ref	0.49 (0.41, 0.57)
Geographic origin		
Central	0.63 (0.32, 1.26)	0.44 (0.33, 0.56)
Northeastern	0.94 (0.47, 1.86)	0.54 (0.42, 0.65)
Western	0.85 (0.38, 1.88)	0.51 (0.36, 0.66)
Khartoum	ref	0.55 (0.43, 0.67)
Geographic residence		
Central	0.62 (0.31, 1.23)	0.43 (0.30, 0.58)
Northeastern	0.85 (0.43, 4.68)	0.51 (0.37, 0.65)
Western	0.62 (0.25, 1.53)	0.43 (0.25, 0.64)
Khartoum	ref	0.55 (0.47, 0.64)

OR: Odds ratio; CI: confidence interval; PP: predicted probability

^a^Models adjusted for age

^b^Arab ethnic group and Khartoum region served as reference groups (ref)

**Table 3.** Frequencies of treatment modalities offered or administered (*N* = 237).

Treatment combinations	*n* (%)
Chemotherapy alone	98 (41)
Chemotherapy, hormone therapy	21 (9)
Chemotherapy, hormone therapy, radiotherapy	1
Chemotherapy, radiotherapy	13 (5)
Chemotherapy, surgery	27 (11)
Chemotherapy, surgery, hormone therapy	10 (4)
Chemotherapy, surgery, hormone therapy, radiotherapy	1
Chemotherapy, surgery, radiotherapy	8 (3)
Hormone therapy alone	16 (7)
Radiotherapy alone	3 (1)
Surgery alone	9 (4)
Surgery, hormone therapy	3 (1)
Surgery, hormone therapy, radiotherapy	2 (1)
Surgery, radiotherapy	1
N/A	24 (10)

**Table 4.** Association between breast receptor status and stage and treatment modality received.

	Receptor status					*p*-value	Staging					*p*-value
	HR+/HER2 +	HR+/HER2-	HR−/HER2+	TNBC	No receptor status		Stage I	Stage II	Stage III	Stage IV	No staging available	
	***n* (%)**						***n* (%)**					
Surgery yes	8 (29)	21 (30)	4 (16)	18 (40)	9 (13)	0.013	6 (40)	98 (26)	25 (28)	3 (9)	17 (25)	0.166
No	20 (71)	49 (70)	21 (84)	27 (60)	60 (87)		9 (60)	25 (74)	63 (72)	29 (91)	51(75)	
Chemotherapy yes	16 (57)	50 (71)	18 (72)	37 (82)	56 (81)	0.094	9 (60)	29 (84)	67 (76)	22 (69)	50 (74)	0.345
No	12 (43)	20 (29)	7 (28)	8 (18)	13 (19)		6 (40)	7 (15)	21 (24)	10 (31)	18 (26)	
Hormone therapy yes	12 (43)	16 (23)	9 (36)	4 (9)	12 (17)	0.004	6 (40)	7 (21)	18 (20)	6 (19)	16 (24)	0.521
No	16 (57)	54 (77)	16 (64)	41 (91)	57 (83)		9 (60)	27 (79)	70 (80)	26 (81)	53 (77)	
Radiation therapy yes	4 (14)	12 (17)	3 (12)	3 (7)	5 (7)	0.319	3 (20)	2 (6)	7 (8)	3 (9)	12 (18)	0.205
No	24 (86)	58 (83)	22 (88)	42 (93)	64 (93)		12 (80)	32 (94)	81 (92)	29 (91)	56 (82)	

HR: hormone receptor, HER2: human epidermal growth factor receptor 2, TNBC: triple-negative breast cancer

**Table 5.** Association between staging and treatment combinations received.

	Stage					
**Treatment combination**	**I**	**II**	**III**	**IV**	**N/A**	**Total**
Chemo	2 (13.3)	17 (50.0)	36 (40.9)	18 (56.3)	25 (36.8)	98 (41.4)
Chemo, Horm	4 (26.7)	5 (14.7)	6 (6.8)	1 (3.1)	5 (7.4)	21 (8.9)
Chemo, Horm, Rad	0	0	0	0	1 (1.5)	1 (0.4)
Chemo, Rad	0	1 (2.9)	4 (4.6)	1 (3.1)	7 (10.3)	13 (5.5)
Chemo, Surg	0	5 (14.7)	16 (18.2)	2 (6.3)	4 (5.9)	27 (11.4)
Chemo, Surg, Horm	0	1 (2.9)	4 (4.6)	0	5 (7.4)	10 (4.2)
Chemo, Surg, Horm, Rad	0	0	1 (1.1)	0	0	1 (0.4)
Chemo, Surg, Rad	3 (20.0)	1 (2.9)	1 (1.1)	0	3 (4.4)	8 (3.4)
Horm	2 (13.3)	1 (2.9)	5 (5.7)	5 (15.6)	3 (4.4)	16 (6.8)
Rad	0	0	2 (2.3)	1 (3.1)	0	3 (1.3)
Surg	3 (20.0)	2 (5.9)	1 (1.1)	1 (3.1)	2 (2.9)	9 (3.8)
Surg, Horm	0	0	2 (2.3)	0	1 (1.5)	3 (1.3)
Surg, Horm, Rad	0	0	0	1 (3.1)	1 (1.5)	2 (0.8)
Surg, Rad	0	0	0	0	1 (1.5)	1 (0.4)
N/A	1 (6.7)	1 (2.9)	10 (11.4)	2 (6.3)	10 (6.3)	24 (10.13)

Chemo: chemotherapy; Horm: hormone therapy; Surg: surgery; Rad: radiation therapy
